# Promising Technologies in the Field of Helminth Vaccines

**DOI:** 10.3389/fimmu.2021.711650

**Published:** 2021-08-19

**Authors:** Dilhan J. Perera, Momar Ndao

**Affiliations:** ^1^Division of Experimental Medicine, McGill University, Montreal, QC, Canada; ^2^Program of Infectious Diseases and Immunity in Global Health, Research Institute of the McGill University Health Centre, Montreal, QC, Canada; ^3^Department of Microbiology and Immunology, McGill University, Montreal, QC, Canada; ^4^National Reference Centre for Parasitology, Research Institute of McGill University Health Centre, Montreal, QC, Canada

**Keywords:** helminth, vaccine, adjuvant, nucleic acid vaccine, recombinant protein vaccine, viral vector, next generation vaccine, next generation vaccinology

## Abstract

Helminths contribute a larger global burden of disease than both malaria and tuberculosis. These eukaryotes have caused human infections since before our earliest recorded history (i.e.: earlier than 1200 B.C. for *Schistosoma* spp.). Despite the prevalence and importance of these infections, helminths are considered a neglected tropical disease for which there are no vaccines approved for human use. Similar to other parasites, helminths are complex organisms which employ a plethora of features such as: complex life cycles, chronic infections, and antigenic mimicry to name a few, making them difficult to target by conventional vaccine strategies. With novel vaccine strategies such as viral vectors and genetic elements, numerous constructs are being defined for a wide range of helminth parasites; however, it has yet to be discussed which of these approaches may be the most effective. With human trials being conducted, and a pipeline of potential anti-helminthic antigens, greater understanding of helminth vaccine-induced immunity is necessary for the development of potent vaccine platforms and their optimal design. This review outlines the conventional and the most promising approaches in clinical and preclinical helminth vaccinology.

## Introduction

Neglected tropical diseases (NTDs) affect approximately one sixth of the world’s population (1). Of these, the group which affects the largest population is the helminths (2, 3). Parasitic worms are some of the most ancient pathogens; eggs from *Schistosoma* for example have been discovered as early as 1200 B.C. in Egyptian mummies (4). Although these worms have been around for millennia, any of them have yet to be eradicated.

**Figure 1:**
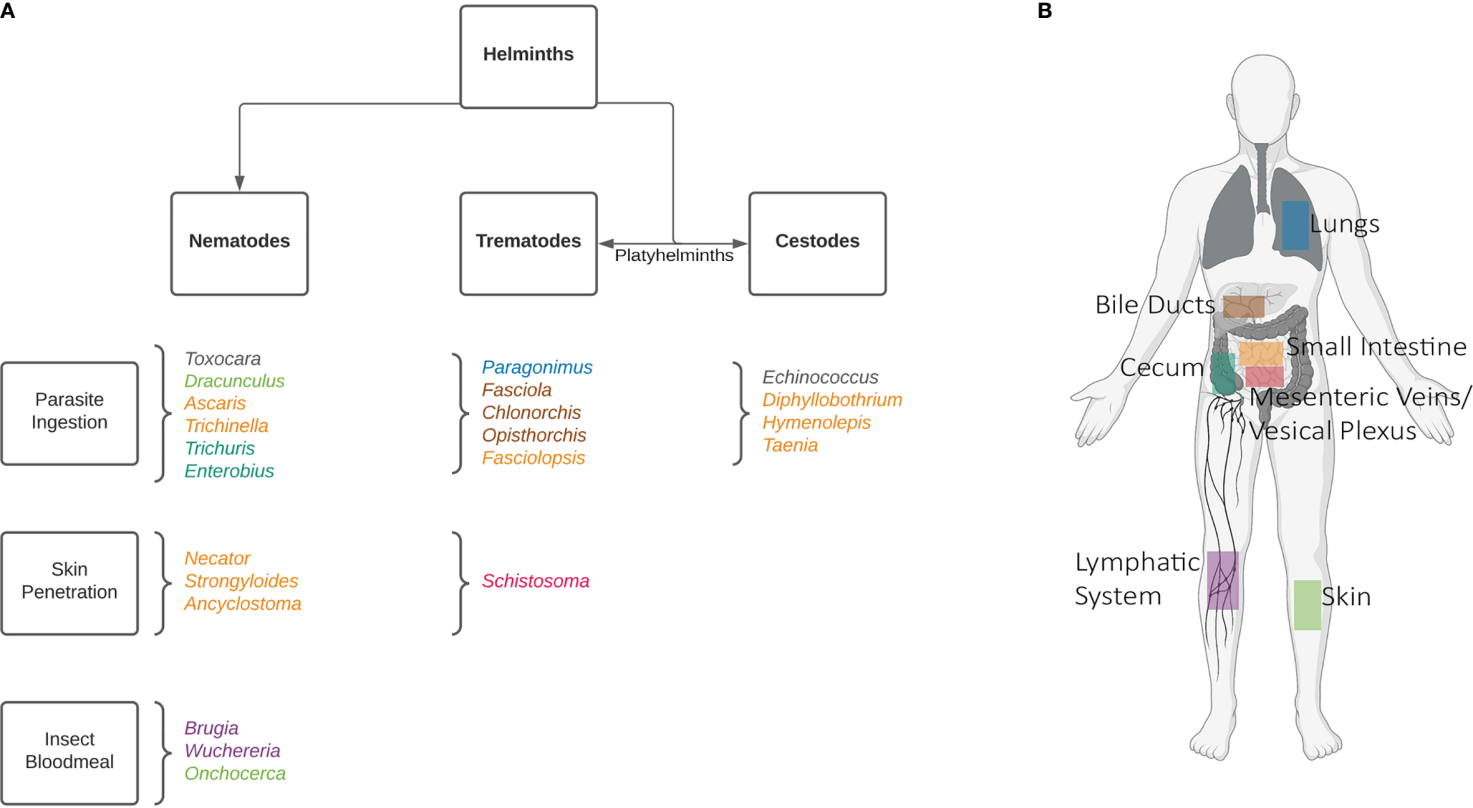
Common human helminths. A non-exhaustive list of human infecting helminths can be found in **(A)** categorized by their morphologies and means of infection. Helminths are named according to genus. To further demonstrate the complexity of these worms’ life cycles their host niches have been shown in **(B)**, where adult worms reside. In the case of *Toxocara*, the life-cycle stops at a larval stage in humans, and can be found in various organs. *Echinococcus* oncospheres are released in the intestines, and hydatid cysts can also develop in various organs. Created with BioRender.com.

Helminths are diverse, comprising over 280 species which can infect humans (5). This number increases dramatically when the one health approach is also considered, including animals. Broadly, these worms are classified into two categories based on morphology: nematodes (roundworms), and platyhelminths (flatworms). The platyhelminths can be further divided into cestodes (tapeworms), and trematodes (flukes). A chart of the most prominent helminths can be found in **Figure 1**. Despite infections being most common among rural communities in tropical and subtropical regions, some of these worms can be found globally.

The prevalence of helminths can be attributed to their extraordinary ability to modulate the immune system. This quality allows them to suppress responses that could result in their elimination, helping them establish chronic infections. Host immunity has developed to limit pathology, resulting in many asymptomatic cases (6) and contributing to the neglect of helminthic infections. This is exceedingly apparent in the case of *Mansonella* spp. (7). However, these parasites can cause a spectrum of disease including debilitating morbidity, while milder cases compromise immunity to other vaccines (8, 9) and incoming infections. In endemic regions, chronic helminth infections lead to increased vulnerability to other pathogens such as HIV (10, 11), malaria (12, 13), and even diseases like cancer (14–17). Further, helminth coinfections with other pathogens or other helminths can make prognoses worse. For example, blood-fluke *Schistosoma* becomes more deadly in Egypt where hepatitis C virus is prevalent leading to severe liver disease (18). Moreover, the nematode *Strongyloides* was shown to reduce monocyte and T cell activation increasing the pathogenicity of tuberculosis infections (19), and human T-cell leukemia virus 1 (20).

Currently, helminth infections are resolved using drug therapy and prevented by various methods including vector control, health education, and programs of water, sanitation, and hygiene (WASH). These efforts have been notable in cases such as soil transmitted helminths (STH) in China (21) and filariasis in Thailand (22) and Sierra Leone (23), among others. Yet in many affected regions despite mass drug administration (MDA) and WASH programs, helminths remain a problem (24, 25) due to low drug efficacies, reinfection, and a lack of other control measures. Additionally, as many helminths are treated with a limited number of drugs, resistance to anthelmintics is emerging for several species (26, 27). Unlike in the case of guinea worm (28), where cases have dropped from 3.5 million in 1986 to 27 in 2020 (29) by community-based education, the elimination of many other helminths can’t be accomplished using singular control measures alone. To reach WHO goals of helminth elimination, various tools (MDA, vector control, education, etc.) should be combined; vaccines make an important addition to this multipronged strategy.

Vaccination has been essential to the excision of several pathogens (30), yet to date there are no anti-helminth vaccines licensed for human use. Helminths are eukaryotic organisms possessing many characteristics which make their targeting by vaccination methods difficult. Helminths are multicellular invertebrates, which exhibit complex life cycles with different life stages, often occupying vectors for transmission, and infecting multiple hosts both intermediate and definitive. Through the study of paleoparasitology we know that helminths, similar to other parasites, have co-evolved with humans (4) and have undergone unique adaptations that allow them to evade the immune system (31), often co-living undetected. These immune evasion techniques are essential to their ability to establish chronic infections.

However, the world of helminth vaccinology is not so dire. Research has shown protection among individuals, although many of these correlates remain unelucidated. In the case of lymphatic filariasis (LF), caused by species *Wuchereria bancrofti*, *Brugia malayi*, and *B. timori*, mathematical modeled studies suggest the emergence of herd immunity in endemic communities (32). More recently, evidence shows that prevalence of infection with LF shares a negative correlation with age. It was found that younger individuals are more susceptible to infection (33), speculating that protective immunity may be developed with time. Similarly, protection from *Schistosoma* spp. has been witnessed over multiple rounds of praziquantel therapy, although immunity takes years and is rarely sterile (34). The proposed mechanism of protection involves the release of cryptic antigens, which are normally hidden to the host immune system, upon parasite death and degradation. After multiple exposures to these antigens, immunity may be developed which target future schistosome infections (35).

Numerous studies and models have additionally shown the phenomenon of concomitant immunity where adult parasites will prevent reinfection to avoid superinfection, protecting both the host and themselves. Penetration from *Echinococcus* oncospheres is immunogenic and leads to significant acquired resistance against egg reinfection (36). This immunity is also utilized by *Schistosoma* spp. (37, 38), although the mechanism of protection remains controversial. A recent review by Buck et al., propose the excretory/secretory molecules and vesicles of adult worms may direct an immune response which targets incoming larval parasites (39). Analogous protection has also been demonstrated in calves (40), supporting the findings that *Dictyocaulus viviparus* larvae are able to induce protective immunity from homologous reinfection (41), limiting host parasite burden. Even female LF worms when subcutaneously implanted are able to partially protect from superinfection in animal models (42–44).

For these reasons, and the discovery and publishing of other correlates of immunity, it is our belief that there is a strong rationale for the development of effective helminth vaccines. Three vaccines for livestock are currently commercially available against *D. viviparus* (bovine lung worm), *Haemonchus contortus* (barber’s pole worm), and *Echinococcus granulosus* (45). These multi-dose vaccines are effective and reduce parasite burden up to 98%, 94%, and 100% respectively. Vaccines for human use are currently in development, with promising constructs in clinical and preclinical trials. This review will summarize various helminth vaccination techniques and compare their efficacy for helminth protection with an emphasis on novel technologies.

## Immune Response to Helminths

The dominant immune response to helminths is widely accepted to be type 2 (Th2) (46), through interactions between the innate immune system, antigen presenting cell (APC) and T helper cell complexes, and the combination of IL4 and IL33. This feature is reflected in many helminth species regardless of their biological niche in the body (i.e., vasculature, intestinal lumen, subcutaneous sites, lymphatic system, etc.), and in most cases coordinated Th2 responses have been demonstrated to protect from parasitic worms (46). Increased levels of classical Th2 cytokines: IL4, IL5, and IL13 have been associated with lower parasite burdens (47, 48) by activating eosinophils, mast cells, alternatively activated macrophages, and antibody defenses like IgE (49–51). Additional cytokines IL6 and IL9 have also been implicated in immunity to filariasis and human whipworm *Trichuris trichiura* (52–54).

Most helminths also invoke immunoregulatory responses by upregulating TGFβ production or releasing parasite-derived TGFβ mimics, to expand regulatory T cell differentiation and promote their persistence. Macrophages and leukocytes have been implicated in IL10 and TGFβ production, downregulating parasite clearing T cell responses and cytokine production (55–57). These regulatory elements enhance parasite survival by leading to the increase of regulatory dendritic cells, regulatory B cells, and alternatively activated macrophages - permitting the development of chronic infections. At first glance the reduction of immune activation may seem to be solely at the cost of the host, yet this IL10 pathway also moderates destructive immune responses, protecting the host from self-damage (58, 59).

**Figure 2:**
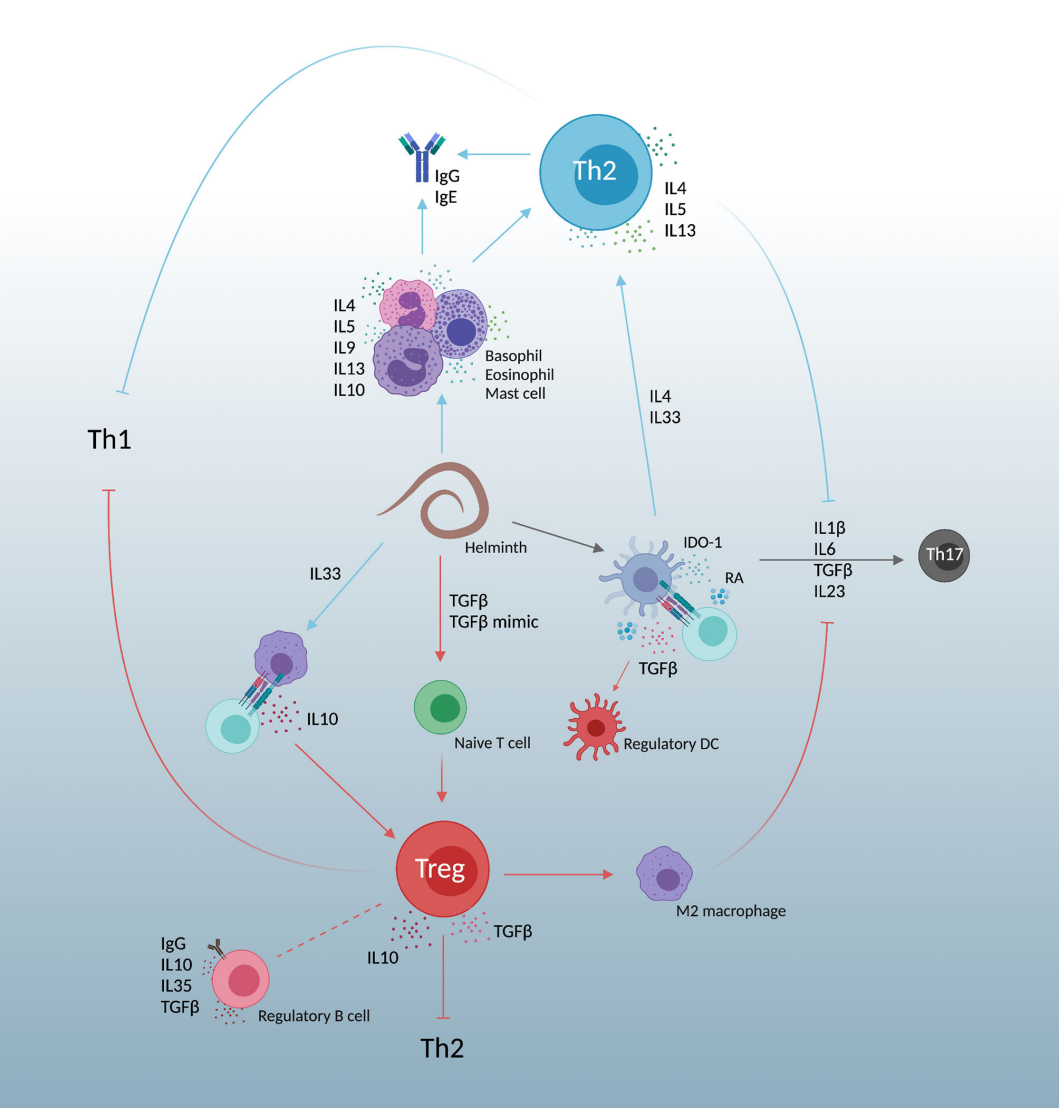
A simplified view of the “modified Th2” response created by helminth infections. Responses are heavily Th2, promoting IL4, IL5, and IL13. Simultaneous expansion of regulatory T cell immunity by host TGFβ and parasite TGFβ mimics dampen Th2 skewing, allowing parasite persistence and decreasing both Th1 and Th17 responses. Created with BioRender.com.

The upregulation of Th2 and regulatory T cell (Treg) responses in helminth infections leads to a “modified Th2” response (60), simplified in **Figure 2**. This suppresses Th1 immunity and is further complicated by Th17 function. Helminths which lead to liver disease tend to increase Th17 cytokines and worsen inflammatory pathologies (61). Dendritic cell derived IL6, TGFβ, and IL23 cause naïve CD4+ T cell differentiation into Th17 cells (62). This differentiation however is dampened by both helminth induced Th2 and Treg responses.

Although it is acknowledged that Th2 responses are important effectors of helminth protection, it is unclear which specific immune mechanisms must be rescued by vaccination. As such, through various vaccination strategies, the search for the ideal anti-helminth immune response continues.

## Irradiated Helminth Vaccines

Similar to attenuated viral vaccines, among the first proposed vaccines for helminths were radiation attenuated. First tested in the 1950s, and leading to the commercial vaccine Dictol^®^, was the live attenuated vaccine against *D. viviparus* in cattle. This vaccine is an oral vaccination with irradiated larvae that are unable to mature into adult worms but survive long enough to stimulate protective immune responses. Sprouting from this research came various vaccine constructs developed using X-ray, γ-ray, UV, and even microwave irradiation against a diverse collection of helminths including but not limited to: LF (63–66), amphistomes (67), STH (68–73), *Fasciola* spp. (74–77), *Toxocara canis* (78, 79), *Trichinella* spp. (80, 81), *Onchocerca volvulus* (82–84)*, Clonorchis sinensis* (85), and *Echinococcus granulosus* (86).

Perhaps the most widely researched irradiated helminth vaccines are against *Schistosoma* spp. Onwards of 1962, researchers have tested irradiated schistosome vaccines (RA vaccines) in animal models of the most clinically relevant species: *S. mansoni* (87–89)*, S. japonicum* (90, 91), and *S. haematobium* (92–95). This vaccination strategy has been referred to as the “gold standard” for years, as it consistently generates high levels of protection against challenge.

Most groups show that protection increases with additional boosting doses of irradiated parasite (70, 81), although the amount of radiation is controversial and may be species specific. In 1986, a study showed that increased levels of γ radiation afforded increased protection from *Fasciola gigantica* (77). In this study, metacercariae were irradiated with 3 and 20-krad and were used to immunize zebu calves. These vaccinated groups gave protection from adult parasites of 77% and 88% respectively, with immunizing radiated parasites only developing into adult worms in the 3-krad group. In contrast, Harrison et al. showed that against *S. haematobium*, cercariae that were given two to three doses of 20-krad radiation were more effective than doses of 3-krad, 60-krad, and much more effective than a single vaccination (95).

The correlates of immunity provided by these vaccines are debated within the literature. In the case of RA vaccines, an importance has been placed on the expression of IFNγ and Th1 immune responses (96–99), despite the protection delivered by Th2 responses for most helminths (65, 72, 83, 100).

Although effective, these vaccines pose challenges as human vaccines for several reasons. They are composed of live parasites which require vectors and experimental models to be grown and maintained. This makes culturing large amounts of helminths impractical. Moreover, the heterogenous nature of eukaryotic worms makes batch manufacturing of a homogenous vaccine impossible. Additionally, in many cases of these constructs, some viable parasites will mature into adult worms and cause patent infections in immunized individuals. Due to logistical and ethical reasons it is unlikely that an irradiated helminth vaccine will be developed for human use. However, the protection mediated by these vaccines further rationalizes the ability to induce protective immunity from parasitic worms, and this research has been used to give insight on the development of promising subunit vaccines.

## Subunit Vaccines and Antigen Selection

To ameliorate some of the challenges of developing radiation attenuated helminth vaccines, many recent strategies utilize subunit vaccines. In this case the most promising components, or antigens, which best stimulate the immune system are administered. By carefully selecting appropriate antigens, vaccine development can be targeted to enhance protection and minimize possible side effects (101). Antigens are habitually identified by proteomic analysis on crude homogenates of helminths and then chosen based on immunogenicity and their ability to stimulate an immune response. In recent years, new technologies have allowed the identification and prediction of antigens using immunoinformatics (102, 103) and *in silico* approaches which use computer software to anticipate T cell epitopes given pathogen genome analysis (104). These antigens can then be isolated from parasites, produced as recombinant proteins, or delivered using innovative vaccine strategies.

## Combining Recombinant Proteins With Novel Adjuvants

To better enhance immune responses, several vaccine efforts have employed the use of novel adjuvants - some of which have not yet been approved for human use. Antigens used as vaccine targets often lack immunogenicity. Helminth antigens, capable of stimulating immune responses, have been known to possess inbuilt adjuvanticity (105, 106). Nevertheless, adjuvants can be used to augment or skew immunogenicity to enhance protective effects. The most common adjuvants used in US vaccines include: formulations of aluminum, AS04, MF59, AS01_B_, and CpG dinucleotides (107). These adjuvants are effective and have demonstrated protective capacities, explaining their use in clinical applications; however, the field of adjuvant discovery is expanding, and several innovative adjuvants are in preclinical trials with promising results.

Many helminth vaccines in clinical development utilize the synthetic TLR4 agonist, glucopyranosyl lipid adjuvant (GLA). Naturally, some preclinical efforts have followed the same path. A recent effort to protect from *S. mansoni* involved adjuvanting the parasitic large subunit of calpain (Sm-p80) with a stable emulsion of GLA (GLA-SE). This vaccine, tested in baboons, demonstrated a female worm specific reduction of 93.45%, with an overall protection of 65.9% (108). Although adult worm reduction is enticing, perhaps a more important metric is the reduction of tissue eggs. *Schistosoma* spp. pathology is induced mainly through egg deposition and Th2 responses against egg antigens, which in support of this vaccine, is reduced by 89.95%. All of these greatly surpass the 40% vaccine standard set forth by the WHO and almost reach a 70% vaccine standard which may be more appropriate in light of recent vaccine efforts (109). GLA-SE has also provided protection from LF in a *B. malayi* mouse model. In this case, a tetravalent vaccine was prepared using the following recombinant antigens: heat shock protein 12.6, abundant larval transcript-2, tetraspanin large extracellular loop, and thioredoxin peroxidase (rBm-HAXT). After the three-dose immunization schedule administered subcutaneously, vaccinated animals displayed high titers of antigen specific IgG in serum and peritoneal fluid, predominating in IgG1 with expansions of IgG2. A significant protection of 88.05% was obtained compared to 79.47% and 78.67% given when rBm-HAXT was adjuvanted with conventional alum and mannosylated chitosan (MCA) respectively. All vaccines tested increased the percentage of central memory T cells (T_CM_) in the spleen, with an increased expression of IFNγ specifically in those cells from the GLA-SE vaccine arm (110). When this vaccine was tested in a non-human primate (NHP) model, despite adding another boosting immunization, vaccine efficacy dropped drastically to 57.14% protection (111). Interestingly, in this different animal model, the immune landscape conferred by this vaccine seems to be more balanced Th1/Th2 *versus* the mouse model where the Th1 bias was apparent in splenocyte expressed cytokines IFNγ and IL2, among others. In the NHP study, T_CM_ cells were found to be expressing more IL4, while effector memory T cells (T_EM_) were found to be contributing the IFNγ. This balanced response was seen in peripheral blood mononuclear cell (PMBC) cytokine expression of IFNγ, IL12p70, IL4, IL5, and TNFa, among others. Despite the drop in parasite burden reduction, antibodies developed in the NHP study were able to mediate protection *via* antibody dependent cellular mediated cytotoxicity (ADCC) by recognizing, covering, and killing L3 larvae *in vitro*.

ADCC has been a proposed mechanism of protection in other helminth vaccines including our work on *S. mansoni* using an adjuvanted Cathepsin B (SmCB). The larval stage of *S. mansoni* passes through the lungs and is vulnerable to antibody and cell mediated effectors. When we combined SmCB with Montanide ISA 720 VG (ISA 720), lung stage protection was reported to be provided by ADCC, specifically through macrophages, natural killer (NK) cells, and CD4+ and CD8+ T cells (112). By conducting *in vitro* larval killing assays with and without cells and sera from immunized mice, it was shown that CD4+ T cells and NK cells were able to significantly increase killing only in the presence of immune sera. The necessity of cells and sera from immunized animals for larval killing makes an argument that protection is mediated by ADCC. In the context of a SmCB and ISA 720 immunized lung, CD4+ T cells and NK cells are key players in parasitic killing, aided by CD8+ T cell and macrophage killing, dependent on antibodies. The resulting protection from this vaccine was approximately 60% with a mixed Th1/Th2 based immune response (113). Seppic-produced Montanide series adjuvants are produced with GMP as well as Montanide ISA 51 VG have been developed as human therapeutics. Among these are many other Montanide adjuvants which are used in veterinary vaccines. ISA 720 has also been used in a vaccine against *Ascaris suum* although protection was higher when the target antigen, As16, was formulated in alum (114). Despite the protection afforded by the Th1/Th2 skewing *S. mansoni* vaccine, in the case of *A. suum* protection was increased with Th2 responses. Other Montanide based vaccines have been developed against *Fasciola* spp. (115–118)*, Schistosoma japonicum* (119)*, Trichinella spiralis* (120, 121), and LF (122) to name a few.

Other vaccines against *Fasciola* spp. have been developed using the adjuvant Quil A. This saponin based adjuvant contains the water-extractable fraction of the South American tree *Quillaja saponaria* Molina and has been used in several veterinary applications (123, 124). With antigens *Fasciola hepatica* Cathepsin L1 (FhCL1) (125), *S. mansoni* 14 kDa (Sm14) (126), and *F. hepatica* peroxiredoxin (FhPRX) (127), and the former three in combination (128), Quil A adjuvanting was unable to significantly reduce parasite burden, although vaccination was able to deliver reductions in pathological events. When a group employed the use of FhCL1 mimotopes (peptides which mimic antigenic epitopes), Quil A was able to reduce parasite burden by 67.17% in sheep (129) and 79.53% in goats (130), suggesting that in some cases choosing appropriate portions of an antigen might be preferable to using whole antigens.

Quil A is an InvivoGen product and has not been developed for human use. This is also the case for AddaVax, a squalene-based oil-in-water nano-emulsion. However, AddaVax is a formulation similar to MF59 which is licensed for human use in 30 countries (131), making it an excellent mimetic for preclinical research. Although in animal models of *A. suum*, when As16 was adjuvanted with AddaVax, there was no protection observed (114), when combined with As37, AddaVax reduced lung larval burden by 48.7% (132). In this context, AddaVax increased both IgG subtypes IgG1 and IgG2c and cytokines IL4, IL5, IL10, IL13, and TNFα. This data corroborates our work combining AddaVax with SmCB in a prophylactic vaccine for *S. mansoni*. We saw robust antigen specific humoral responses of both IgG1 and IgG2c. In addition, we observed a significant increase in systemic CD4+ and CD8+ T cells expressing IFNγ along with a splenic immune landscape which skewed more Th2 and anti-inflammatory than when SmCB was combined with ISA 720 and sulfated lactosyl archaeol archaeosomes. Overall, this mixed Th1, Th2, and anti-inflammatory response led to both significantly reduced visual pathology in vaccinated mouse livers and egg granuloma sizes. Moreover, this vaccine reduced parasite burden by 86.8%, 78%, and 83.4% in adult worms, hepatic eggs, and intestinal eggs respectively (133). The only helminth vaccine tested within the last 5 years using MF59 is one developed for *Onchocerca volvulus* which conferred 87% protection in mice when adjuvanting the *O. volvulus* antigen OvRAL2 (134). Although MF59 is largely used in influenza vaccines, the protective responses of AddaVax seen in parasitic models may warrant its application to helminth vaccinology.

*S. mansoni* vaccines have been in development for over 30 years and recent work has been conducted using the adjuvant adaptation (ADAD) system originally developed in 2004 for *Fasciola hepatica* (135). This vaccination system involves two subcutaneous injections. The first “adaptation” immunization contains a combination of synthetic aliphatic diamine and saponins emulsified in a non-mineral oil. Five days later, a second immunization is given with the same elements including antigen. A three-dose vaccine developed using predicted B and T cell epitopes of an *S. mansoni* kunitz-type serine protease inhibitor was tested using the ADAD system. Both epitopes used conferred protection, however the T cell epitope delivered a slightly higher parasite burden reduction of 91% in female adult worms than the B cell epitope vaccine (89% reduction). Interestingly, both these vaccines were sex specific and male adult worms were unaffected. Contrary to the worm reduction, the B cell epitope delivered a higher reduction in gut liver eggs (77-81%) compared to the T cell epitope vaccine (57-77%). Still, both had a reduced number of egg-induced granulomas (136). A second effort was also tested against *S. mansoni* using the ADAD vaccination system. Two vaccines were developed comparing recombinant protein expression systems, both of them two-dose, targeting *F. hepatica* fatty acid binding protein [Fh15 (*E. coli* expressed) and Fh15b (baculovirus expressed)] which show a 44% identity to Sm14 in clinical trials as a *S. mansoni* vaccine target. This study demonstrated that when expressed in *E. coli*, Fh15 delivered higher protection from schistosomiasis than when expressed by baculovirus. This finding supports the hypothesis that post-translational modifications by different expression systems can impact the immune response elicited by recombinant antigens, as this group found that delivering a baculovirus expressed Fh15 resulted in an impairment of the humoral response (137). The *E. coli* expressed Fh15 vaccine reduced parasite burden by 64%, 69%, 58%, 67%, 61%, and 77% in adult worms, female worms, male worms, hepatic lesions, and eggs per gram of liver and intestines respectively. Despite this protection being lower than the protease inhibitor ADAD vaccine, the reality of a schistosome vaccine targeting a *Fasciola* antigen could mean cross protection from both helminths, which is incredibly alluring for co-endemic regions.

Although the ADAD system seems to be effective, the advancement of this vaccine strategy may prove to be infeasible as each immunization consists of two injections five days apart. The first vaccine described would result in six necessary injections, and the second would be four. In endemic regions, vaccine compliance and the lack of infrastructure will challenge the ability to properly vaccinate the population. Current helminth vaccines use three immunizations as a standard, however effective vaccine strategies requiring fewer boosting immunizations should be explored.

## Nucleic Acid Vaccines

In recent years, the push for nucleic acid vaccines have become more prominent. To our knowledge there have been no RNA vaccines developed against helminthic infections, however DNA vaccines have been tested since the early 2000s. Genetic vaccines deliver antigen RNA or DNA which are then translated within the host for *in vivo* antigen expression. Internal delivery of antigens using DNA is compelling as it is easy to manufacture and has been demonstrated to induce both humoral and cell-mediated immune responses in animals (138, 139). DNA is also stable at ambient temperatures (140) which is highly practical for use in endemic and rural regions.

A common obstacle of DNA vaccines seems to be a lack of immunogenicity when used in humans, which groups have been attempting to ameliorate through the use of molecular adjuvants and advanced DNA vector design (141, 142). DNA vaccines may also pose safety risks associated with biodistribution and persistence, as well as the potential for plasmid-based vaccines to be integrated into the microbiome genome. To subside some of these fears, Liu et al. show that although their *S. japonicum* plasmid-based DNA vaccine can be found in every tissue site tested, it was successfully cleared by day 120 (143). Additionally, after vaccination with their plasmid containing a hygromycin resistance gene, intestinal microfloras were unable to grow on hygromycin containing plates, demonstrating that the microbiome of vaccinated animals was not found to uptake the plasmid. To confirm, they also ran a PCR for the hygromycin gene and their gene of interest on DNA extracted from intestinal and excremental samples and obtained negative results. To date there are no DNA vaccines approved for human use, although four are licensed for veterinary use (144) and there are over 600 clinical trials that focus on DNA vaccination registered in the USA.

In a preclinical context, DNA vaccines have been developed against several helminths including *Fasciola hepatica* (145), hookworm (146), and *Schistosoma* spp. (147–150) for example. However, many of these vaccines deliver lackluster parasite burden reductions, barely reaching 50%.

Several efforts have been made to produce a DNA vaccine against *Trichinella spiralis*. In 2013, Tang et al. published their data of a vaccine which reduced parasite burden by 37.95% (151). This vaccine encoded two antigens [*T. spiralis* macrophage migration inhibitory factor (TsMIF) and cystatin-like domain protein (Ts-MCD-1)] and was delivered in two doses. They found their vaccine to stimulate Th1 responses, increasing IFNγ with no significant changes to IL4 and IL5 expression, similar to the results of a *S. mansoni* DNA vaccine which only reduced parasite burden by 30% (147). In the same year, a DNA vaccine delivered in 3 doses alongside recombinant protein (Ts87) was able to further reduce parasite burden by 43.8% (152). This vaccine increased both Th1 and Th2 immune responses with cytokine expression increases of IL2, IL4, IL6, and IFNγ. It is interesting to note that optimal antigen selection is crucial for protective immunity from helminths. Although the TsMIF+Ts-MCD-1 vaccine was only partially protective, a DNA vaccine expressing *T. spiralis* 43 kDa and 45 kDa glycoproteins was able to confer protection of 75.9% (153). This vaccine also deployed a mixed Th1/Th2 immune response alike the Ts87 vaccine, without the need for additional recombinant proteins. The Ts43+Ts45 vaccine showed increases of IFNγ, IL4, and IL10, but more striking was the increase in the percent of B220+ B cells when compared to both single antigen DNA vaccines and the PBS control. These data support the idea that both Th1 and Th2 arms of immunity can work synergistically to protect from parasitic worms.

The most significant parasite burden reductions afforded by DNA vaccines can be seen in their use against *B. malayi* and LF. Gupta et al. has demonstrated the protective efficacy of two DNA vaccines, both of which use a heterologous DNA prime and recombinant protein boost strategy targeting a myosin gene. An initial endeavor gave two immunizations of DNA followed by two protein boosts adjuvanted by Freund’s incomplete adjuvant (FIA). This vaccine reduced parasite burden by 75.3% and showed a 78.5% reduction in microfilarial density in the blood (154). Antibody responses were shown to kill L3 larvae, and cytokines IL2, IFNγ, TNFα, IL12, IL4 and IL10 were increased after immunization and maintained through challenge. This immunogenicity was increased when their DNA vaccine was delivered along with CpG dinucleotides and in replacement of FIA. This 4-dose vaccine was now found to reduce parasite burden by 84.5% with similar cytokine expression and the additional proliferation of CD4+ T cells, CD8+ T cells, and CD19+ cells. Likely, the increase of dendritic cell (DC) activating markers and T cell associated markers (CD40, CD80, CD86) observed on DCs from vaccinated animals combatted the APC dysfunction and lack of T cell responsiveness common to filarial infection (155, 156).

DNA vaccines typically promote Th1 immune responses. Although these show promise in models of LF, other helminths may be better targeted by alternative methods of vaccination. This can be seen more explicitly in the Sm-p80 vaccine which has been tested in baboons against *S. mansoni* delivered both by DNA vaccines and as adjuvanted protein. The Sm-p80 vaccine has been in development for over a decade, optimizing through various vaccine platforms and adjuvant combinations (157). The most significant protection data from the Sm-p80 DNA vaccines were obtained when a DNA vaccine was boosted by recombinant protein adjuvanted with CpG dinucleotides, which reduced parasite burden by 47.34% (157). This reduction is less enticing than that of their adjuvanted recombinant protein vaccine using GLA-SE (65.9%) (108). Zhang et al. conducted RNA-sequencing on PBMCs, spleen cells, and lymph node cells from baboons vaccinated with their various Sm-p80 vaccines to determine functional immune profiles from each. The DNA vaccine presented a relative Th1-mediated immune response with 8.41% of its differentially expressed genes in PBMCs relating to the TLR9 signaling pathway. This was reflected by a predicted deactivation of Th2 pathways in spleen cells and iCOS-iCOSL signaling in both spleen cells and PBMCs. This downregulation of Th2 immunity may have diminished protective correlates, as in previous reports, Sm-p80-mediated protection seems to be enhanced by antibody responses (108, 158).

Supported by a hypothesis by Versteeg et al. (159), an interesting avenue of research will be the development of RNA vaccines for helminths which, given the efficacy of their COVID-19 counterparts, can be expected in the near future. Their versatility and simple means of production made mRNA vaccines a top contender for prophylactic SARS-CoV-2 vaccines and were exploited by the two earliest vaccines made available by the FDA for COVID-19 emergency use (160, 161). RNA vaccines are shown to stimulate potent and safe immune responses in animals (162) and humans (163–165). Although their stability requires a cold-chain, mRNA vaccines have a low-cost manufacturing process, and unlike DNA vaccines, they remain outside the host cell nucleus making them an attractive vaccine vector worth exploring.

## Viral Vectored Vaccines

Since the discovery of vaccines and the efficacy of live attenuated virus vaccines to protect from their wildtype counterparts, the concept of using viruses to fight other infectious agents has evolved over time. Around the introduction of nucleic acid vaccines like DNA and RNA vaccines, delivering genetically modified viruses arrived as another vaccine platform. Viral vectored vaccines utilize the natural infectivity of viruses and their “life” cycle, using host machinery to translate their own genetic elements and incorporated antigens. By using infectious agents to deliver vaccine targets potent cellular responses can be elicited, specifically CD8+ cytotoxic T cells. As in the case of nucleic acid vaccines, since vaccine encoded genes are expressed intracellularly, antigens can be processed and presented on class I major histocompatibility complexes (MHC-I) of APCs. These approaches may be favoured for diseases where cell-mediated immunity can significantly enhance protective responses afforded by humoral immunity or in those cases where antibody production alone is insufficient.

Viral vectored vaccines have been developed against Lassa fever (166), HIV (167), malaria (168), taeniasis (169), and countless others. As each viral vectored vaccine can utilize a different virus, each of their mechanisms of antigen presentation and immune stimulation will vary according to the nature of the virus used. Commonly used viral vectors are adenoviruses, pox viruses, and vaccinia viruses because they are well characterized, each with their own unique features. Although many others have been exploited as vectors, only a few will be touched upon here.

One of the largest limitations to viral vectoring is neutralizing antibodies to the vector from previous viral infections. Vaccine priming doses can also produce neutralizing antibodies which may render boosting immunizations useless. To circumvent this problem, despite groups showing robust T cell responses regardless of the presence of neutralizing immunity (170), heterologous prime-boost strategies, varying vaccine immunization routes, and the use of viruses which do not circulate in target populations, have been employed.

Several groups have utilized the antigen EG95, a protective vaccine target for *Echinococcus granulosus*, demonstrating its expression using goatpox virus (171), morbillivirus (172), and on the surface of orf virus (173, 174). Protective benefits of only one *E. granulosus* viral vectored vaccine have been published to date, and this was in a vaccinia virus vector (175). In this model, mice were immunized with 10^8^ plaque forming units (PFU) of recombinant vaccinia virus intranasally. Some groups of mice were given a boosting immunization of either recombinant virus or recombinant EG95 prepared with alum and delivered intraperitoneally. The reverse was also tested, where adjuvanted antigen was the priming immunization for a recombinant virus boost. Analysis of EG95-antibody responses showed the highest titers of immunoglobulin in mice which received virus prior to adjuvanted protein, followed by the group administered the reverse. Protective capacity was demonstrated *in vitro* using an oncosphere killing assay. In this experiment, mouse anti-sera were applied to oncospheres and the highest dilution in which killing was observed was reported. The group in which the highest dilution of sera provided killing was the group first immunized by virus followed by adjuvanted protein. The trend follows that the amount of antigen specific immunoglobulin may be correlated with protection, as those groups with higher titers observed killing at higher dilutions.

The earliest viral vector for *Schistosoma spp*. developed for *S. mansoni* showed no antigen specific antibody response or protective efficacy (176); however, several have since been deployed as a vector for *S. japonicum*. In 2010, data were published on a pseudorabies virus (PRV) expressing both *S. japonicum* fatty acid binding protein and 26 kDa glutathione-S-transferase (177). This vaccine was administered in two doses and increased IL-2 expression from stimulated splenocytes when compared to the negative control. The virus expressing both antigens even significantly increased IFNγ production higher than either viral vaccine expressing single antigens. The protection they observed in mice (39.3% reduction of worms) was less than that of sheep (48.5% reduction of worms), inferring the importance of the animal model used.

Attenuated pseudorabies viruses have been developed against a large number of infectious diseases and have been comprehensively reviewed (178). Although in some cases PRV vectored vaccines have been effective, groups have shown faster antibody responses and recruitment of cell-mediated immunity using adenoviral vectors (179). This schistosomiasis protection delivered by PRV was inferior to another group which expressed *S. japonicum* triosephosphate isomerase using a human adenovirus serotype 5 vector.

An initial study in mice looked at the immune responses and protective efficacy elicited by various administration routes of a recombinant adenoviral vector including: intramuscular (IM), subcutaneous (SC), and oral administration. Vaccines were administered in three doses, each giving 10^8^ PFU of virus. Oral immunization did not result in a humoral response or significant protection. Although robust antibody titers to antigen were observed in the IM and SC routes of administration at the study endpoint, the question of neutralizing antibodies was not addressed, and humoral responses were not measured throughout immunization. Interestingly, the route of administration caused a shift in the isotype of IgG expressed. SC immunization led to an expansion of IgG1/IgG2c, whereas IM immunization favoured IgG2c. This immune skew was reflected in ELISPOT data from stimulated splenocytes. Splenocytes from mice in the subcutaneously vaccinated group had a higher proportion of IL4 secreting cells to IFNγ, and in the intramuscular group the opposite was observed. Although both routes delivered protection from infection, the intramuscular or Th1 skewed response may be more desirable giving 54.92% reduction compared to the SC 37.50% (180). Protection was further increased when their recombinant adenovirus was delivered in a heterologous prime boost strategy using recombinant protein adjuvanted with FIA. This vaccination method promoted expansion of both antigen specific IgG1 and IgG2c isotypes and reduced parasite burden by 72.09% in adult worms compared to the recombinant adenovirus alone and adjuvanted protein arms, which reduced adult worms by 50.59% and 26.67% respectively (181).

This wave of viral vectoring in vaccinology has been used in many preclinical applications and has expanded into human use in the past years (182–184). Other viral vectors of interest which may be considered include cytomegalovirus, vesicular stomatitis virus, and measles virus due to their large carrying capacities (185), and Newcastle disease virus due to the lack of preexisting virus immunity in human populations (186). Although their use in helminthology is lean, viral vectored vaccines allow for enhanced immunogenicity when compared with other genetic vaccine vectors and should be further investigated.

## Helminth Vaccines in Clinical Trials

To date the only helminth vaccines in clinical trials have been developed using recombinant protein technology, generally with adjuvants to enhance immunogenicity as summarized in **Table 1**. These vaccines showed promising data in preclinical studies and have been largely safe and immunogenic in humans. The one exception being the Na-ASP-2 vaccine for human hookworm. Due to the generalized urticaria witnessed with this vaccine (195), hookworm vaccine efforts have shifted to other antigens such as Na-GST-1 and Na-APR-1.

**Table 1:** Helminth vaccines in human clinical trials.

Target Antigen	Adjuvant	Doses	Pathogen	Phase	Ref
Glutathione-s-transferase (Na-GST-1)	Alhydrogel	3	Hookworm	1; complete	(187–190)
	Alhydrogel + CpG				
	Alhydrogel + GLA-AF				
Aspartic protease (Na-APR-1)	Alhydrogel	3	Hookworm	1; complete	(191)
	Alhydrogel + GLA-AF				
Na-GST-1 + Na-APR-1	Alhydrogel + GLA-AF	3	Hookworm	1; complete	(192, 193)
L3 larvae		3	Hookworm	N/A; complete	(194)
Ancyclostoma-secreted protein (Na-ASP-2)	Alhydrogel	3	Hookworm	1; complete and halted	(195–198)
Glutathione-s-transferase (Sh28GST)	Alhydrogel	4	*Schistosoma haematobium*	3; complete	(199, 200)
	Alum	2,3		1; complete	(201, 202)
Sm14	GLA-SE	3	*Schistosoma mansoni*	2/3; ongoing	(203)
				2; complete	(204, 205)
				1; complete	(206, 207)
Tetraspanin (Sm-TSP-2)	Alhydrogel	3	*Schistosoma mansoni*	1/2; recruiting	(208)
	Alhydrogel + AP 10-701			1; complete	(209)
	Alhydrogel + GLA/AF			1; complete	(210, 211)

Currently, the only completed phase 3 trial with reported efficacy data is the vaccine for urinary schistosomiasis. Unfortunately, this 4-dose vaccine regimen proved to be ineffective in providing sufficient protection from *S. haematobium* when looking at the delay in reinfection between experimental groups (199). The authors hypothesize that an expansion of antigen specific IgG4 hinders protective responses of IgG3, despite the previously shown protective effects of IgG4 in *S. haematobium* reinfection (212). Perhaps more likely is their explanation that their efficacy readout was suboptimal, as they were unable to visualize differences in infection intensity in the vaccine group *versus* the control. Despite this lack of efficacy, the confirmed safety of the Sh28GST vaccine in humans and the availability of other promising vaccine constructs in preclinical testing suggest the probability of a protective helminth vaccine in the foreseeable future.

Although not a vaccine for helminths, the furthest progressed human parasite vaccine is the Mosquirix™ (RTS,S/AS01) vaccine which was given a favorable opinion by the European Medicines Agency (EMA) and has been authorized by the National Regulatory Authorities of Ghana, Kenya, and Malawi for use in pilot areas. It combines the central repeat region of the circumsporozoite protein of *Plasmodium falciparum* onto a hepatitis B surface antigen as a carrier, with the adjuvant AS01 and was recently reviewed (213). Within the countries included in the phase 3 trial, RTS,S/AS01 prevented 39% of malaria cases and 29% of severe malaria over four years (214). Although protection can’t be compared to helminths, since malaria is an intracellular protozoan parasite, the success of RTS,S/AS01 gives hope to other parasitic vaccine efforts.

## Helminth Vaccine Induced Protection

The central dogma around helminth protection has encompassed Th2 immunity for decades. This includes cytokines IL4 and IL5, immunoglobulins, and eosinophils. IL4 is an important mediator of Th2 cell differentiation and the activation of the class switching mechanism of B cells to produce IgE, whereas IL5 is a potent growth and survival signal for eosinophils. Eosinophils are correlated with helminth infections (215); although their protective effects are unclear (216, 217), *in vitro* studies have demonstrated helminth killing in models of *Schistosoma* (218, 219) and *Strongyloides* (216). Yet recent efforts are finding effector mechanisms of cell mediated immunity providing protection from various helminth species. Although the Th2 response clearly drives protection in many models, several of the most promising vaccine candidates mentioned in this review tend to elicit both Th1 and Th2 arms of immunity, as summarized in **Table 2**.

**Table 2:** Summary of promising helminth vaccines.

Vaccine Platform	Parasite	Target Antigen	Doses	Parasite Reduction	Animal Model	Immune Skew	Ref
Adjuvant (QuilA)	*Fasciola hepatica*	Cathepsin L1 mimotopes	2	79.5% worms	Goats	Th1/Th2	(130)
Adjuvant (cholera toxin B subunit) intranasal	*Trichinella spiralis*	Serine protease	3	71.1% worms	BALB/c mice	Th1/Th2/mucosal IgA	(220)
				62.1% muscle larvae			
Adjuvant (GLA-SE)	*Schistosoma mansoni*	Sm-p80	4	65.9% worms	Baboons	Th2	(108)
				91.4% liver eggs			
				88.8% intestinal eggs			
Adjuvant (ADAD)	*Schistosoma mansoni*	B-cell epitope of Serine protease inhibitor	3	89% female worms only	BALB/c mice	Th2	(136)
				77% intestinal eggs			
				81% liver eggs			
Adjuvant (AddaVax)	*Schistosoma mansoni*	Cathepsin B	3	86.8% worms	C57BL/6 mice	Th1/Th2/anti-inflammatory	(133)
				78% liver eggs			
				83.4% intestinal eggs			
Adjuvant (GLA-SE)	*Brugia malayi*	Tetravalent fusion protein	3	88.1% larvae	BALB/c mice	Th1/Th2	(110)
DNA/Adjuvant (CpG) prime, Protein/Adjuvant (CpG) boost	*Brugia malayi*	Heavy chain myosin	4	84.5% larvae	BALB/c mice	Th1	(221)
DNA	*Trichinella spiralis*	Co-administered Ts43 and Ts45	3	75.9% muscle larvae	BALB/c mice	Th1/Th2	(153)
DNA	*Schistosoma japonicum*	2 Co-expressed bivalent fusion proteins (tetravalent)	1	70.8% worms	BALB/c mice	not determined	(222)
				60.7% liver eggs			
Adenovirus prime, Protein/Adjuvant (Freund’s incomplete) boost	*Schistosoma japonicum*	Triosephosphate isomerase	4	72.1% worms	BALB/c	Th1/Th2	(223)
				72.1% liver eggs			

Helminths are complicated eukaryotes which have evolved to promote regulatory T cell responses, dampening Th2 type immunity and allowing their chronic persistence. While vaccines may strive to boost Th2 responses, they should maintain Treg responses as regulation plays a vital role in reducing parasite driven pathology. These responses are especially important in schistosomiasis where Tregs protect from immunopathological damage (224–226).

From our review, we believe that the contribution of the type 1 response may be underappreciated and rescued by vaccines to target these worms, especially at vulnerable larval stages. In the case of *Echinococcus*, Th1 responses were shown to be protective (227), and in *Schistosoma* mouse models, IL10 and IL12 knockout mice develop unchecked Th2 responses and exhibit significant mortality in the chronic stages of infection (228). Additionally, a key cytokine of the Th1 response, IFNγ, plays an important role in protection from filariasis (229). Although in natural infection models the immunity afforded by unspecific Th1 responses is diminutive, protection in vaccine models is more pronounced through the expansion of focused cell mediated immunity and Th1 cytokines. It is possible that a carefully balanced immune response, eliciting multiple immune mechanisms directed at vital parasitic molecules, could be the key to protection from helminthic infections.

As worms mature through multiple life cycle stages within the host it is also reasonable that these various facets of immunity can act at different points in time. Where Th2 immunity is often highly effective against adult worms, Th1 and innate immunity may better target juvenile stages of worms travelling through skin and mucosal sites. The immune system’s first responder to invading pathogens is the innate immune system. The first cells to encounter helminths are macrophages, dendritic cells, and other APCs.

Besides upregulating Th2 responses and increasing IL4, IL5, and IgE, other innate immune cells which may provide direct helminth protection are neutrophils and NK cells. Neutrophil extracellular traps (NETs) are web like chromatin structures and are known to protect against large pathogens (230). Research into NETs in helminthic contexts has shown that they negatively impact the fitness of hookworm larvae after skin penetration (231). NETs are also able to trap *Strongyloides* larvae *in vivo*, potentially making them vulnerable to neutrophil, eosinophil, and macrophage killing seen *in vitro* (232). Neutrophils may be an interesting target for other helminths which spend time passaging through skin sites, such as *Schistosoma* and *Onchocerca*. NK cells are not well studied in helminth vaccines, and despite their relation to the innate immune system, there is evidence that these cells can be long lived and acquire antigen specific memory (233, 234). Although their importance to the protection of an *Ostertagia* cattle vaccine is unknown, this vaccine induced NK cell activation (235). These data are reminiscent of previous work using recombinant *Onchocerca volvulus* ASP-1 which induced a dominant IFNγ response, likely produced by activated NK cells (236), albeit NK cells can also act through ADCC and kill *via* degranulation. NK cells may recognize antibody-sequestered parasite and release perforin, granzyme, and granulysin, the latter two of which are upregulated in vaccinated animals against *Ostertagia* (237). Helminth vaccines may work to ramp up innate immune cells, as seen in Bacille-Calmette-Guerin vaccination (238), despite their nonspecific nature.

**Figure 3:**
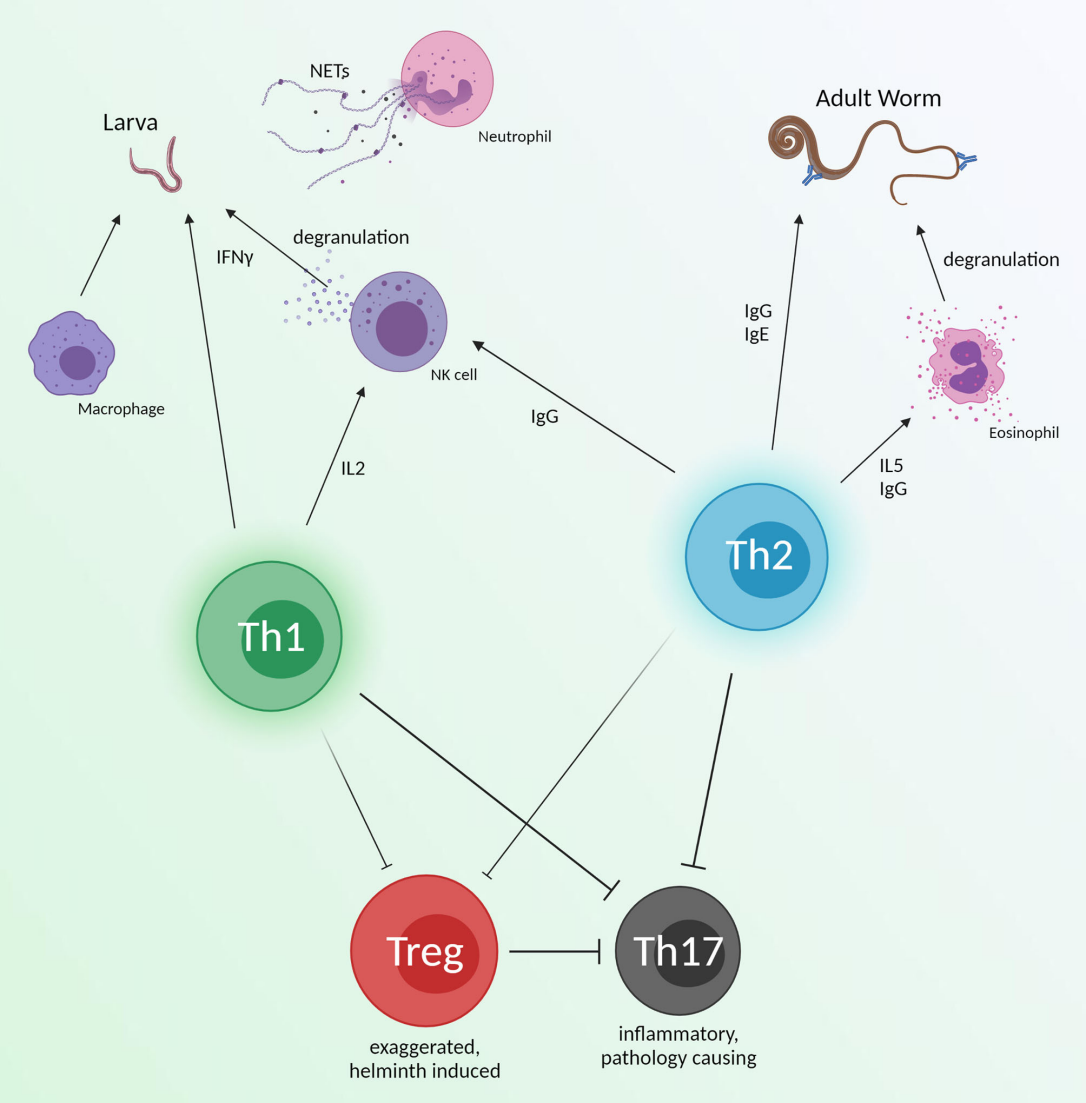
An overview of immune effectors which have shown helminth killing. These responses are broad and could be an ideal immune response for vaccines to emulate. Th1 and Th2 responses may work synergistically with innate immunity to directly target juvenile and adult worms. Simultaneously, helminth induced Treg responses will be diminished, but still prevent inflammation from Th17 function. Created with BioRender.com.

It is difficult to concretely attribute immune effectors of helminth protection provided by vaccines, although a collection of some protective responses can be seen in **Figure 3**. Despite many studies seeking to identify protective mechanisms in infection models, most helminth vaccine studies give only broad descriptions of the conferred immune landscape and systemic immune cell responses after vaccination. A few groups have looked more in detail at vaccine induced immune mechanisms. However, more emphasis should be placed on immune effector knockouts and passive transfer experiments. If we are to find the necessities for helminth protection, we must not only look at general immunogenicity but hone in on specific responses which are indispensable for helminth killing.

## Other Considerations

As briefly mentioned here, targeting multiple antigens in vaccines can sometimes increase protection efficacy. In a tetravalent DNA vaccine for schistosomiasis, parasite burden was reduced by 70.8% in adult worms compared to the 52-53% worm reduction of its divalent equivalents (222). A similar bivalent DNA vaccine was tested before this. Although protection from *S. japonicum* reached only 58.97% in their most effective vaccine, this group showed higher protection than that of their monovalent DNA vaccines (239). The inclusion of multiple antigens also opens an avenue for cross protection against multiple helminth species. This is increasingly tempting as many regions are co-endemic for several helminths (240). Additionally, vaccine parameters including routes of administration (IM, SC, intradermal), specific dosages (50, 100, 200 μg/mouse), and different numbers of immunizations (1, 2, 3) were tested. These parameters should be considered as each variable can change the elicited immune response (241). The importance of vaccine schedule testing is supported by a recombinant adenovirus *S. japonicum* vaccine which showed higher parasite burden reduction when administered in two doses *versus* three (242).

An important caveat learned from the Na-ASP-1 vaccine is the possibility of IgE hypersensitivity in individuals previously exposed to helminths. As parasitic worms are known for their Th2 and IgE responses (243), it is important to assess allergic immune responses against target antigens to avoid vaccine-induced adverse events. As seen in the case of Na-ASP-1, the cross-linking of vaccine induced antibodies with those from natural infection can lead to detrimental responses such as significant histamine release (195). Given the prevalence of helminth infections in endemic regions, it would be operationally difficult to ensure each vaccinated individual is without a potentially cross-reactive previous infection. Instead, antigens which are protective but not recognized by preexisting IgE should be considered, and vaccine candidates should be evaluated for their antigen-specific allergic-type responses.

It is an interesting idea to challenge current vaccination standards. For example, adjuvanted protein vaccines are generally administered IM or SC. However, a *T. spiralis* vaccine targeting a serine protease was administered intranasally with cholera toxin B subunit. This group was able to reduce parasite burden by 71.1% and observed visually shorter worms in vaccinated animals. Most notable about intranasal administration of this vaccine was, in addition to eliciting an antigen specific IgA response in the serum, it induced a systemic mucosal response generating specific IgA in the intestines (220) – which may be increasingly relevant for this gut dwelling parasite. However, in the pursuit of novel approaches we must acknowledge regulatory agencies such as the FDA (USA), BRDD (Canada), MHRA (UK), and EMA (EU). It would be beneficial to choose vaccine technologies which are likely to progress into human trials. For example, vaccines utilizing Freund’s adjuvant are interesting as proof of concept, however they will never be progressed into clinical applications. Novel vaccine development is further complicated in the current era of vaccine hesitancy given the anti-vaccine movement.

Additionally, helminth vaccines are primarily developed for low- and middle-income countries. Although the significant portion of the world’s population is at risk of helminth infection, there is very little commercial potential to develop these vaccines for a population which can’t afford them. It is important to consider production costs when developing helminth vaccines since these infections lack the financial incentive which surrounds many other infectious diseases (i.e.: HIV/AIDS, COVID-19).

## Conclusion

By no means are the platforms discussed here an exhaustive list of those in development for helminth vaccines. In recent years there have been many others employed including: nanoparticles (244), virus-like particles (245), plant-based vaccines (246), as well as bacterial vectored vaccines similar to our own effort (247).

**Figure 4:**
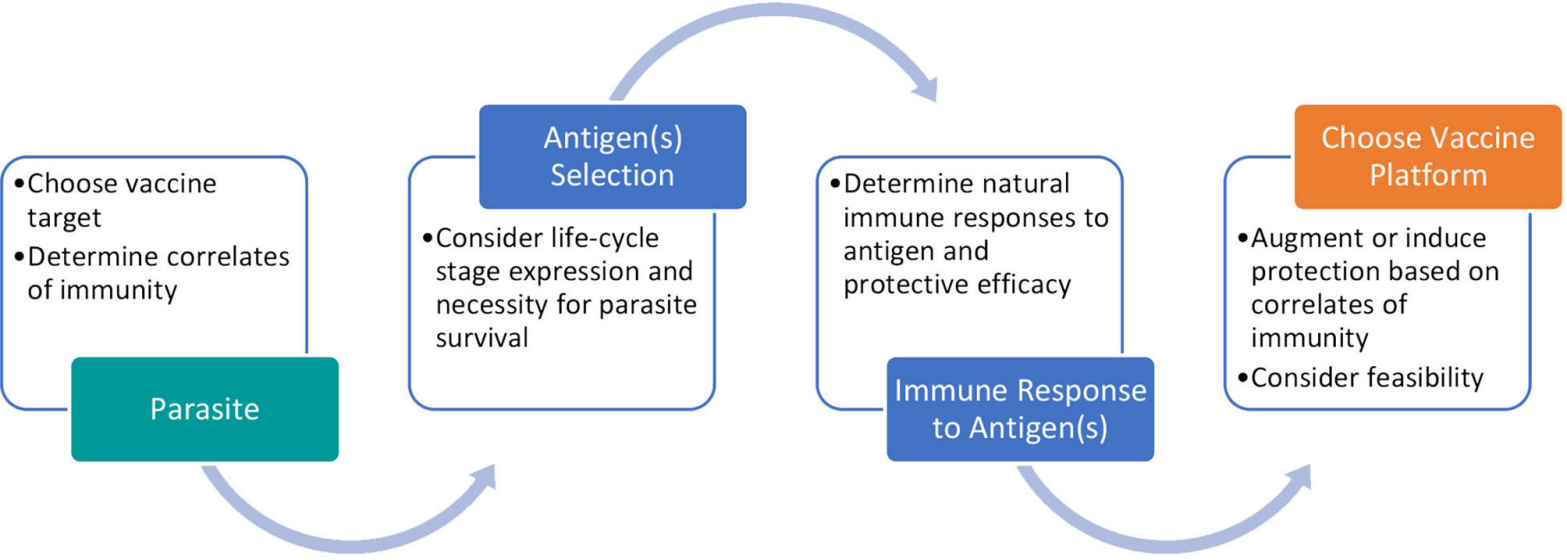
Process for developing a promising helminth vaccine. Our proposed pathway involves careful antigen selection and vaccine platform consideration to reflect protective correlates of immunity.

During the vaccine development process, we propose to follow the path described in **Figure 4**. Determining the most effective platform for helminth vaccination is a convoluted question as each has been shown to have protective efficacy in different models. Instead we suggest researchers start by determining the protective correlates of immunity associated with the targeted helminth(s). Once an antigen is determined, by assessing its necessity to parasite survival and protective efficacy, a novel vaccine platform can be utilized to induce or increase protection.

Each vaccine platform discussed brings forth its own set of positive and negative attributes, each of them when combined with an antigen may elicit unique features of the immune system. Some commonly observed advantages and disadvantages of the platforms discussed are described in **Table 3**, considering each platform independently. Adjuvanted recombinant protein vaccines are well studied and have been shown to be highly effective in animal models. However, recombinant protein expression systems and adjuvants can be expensive, which may not be ideal for a vaccine that is geared for use in the global South. With the authorization of vaccines utilizing mRNA-based and viral vectored technology catalyzed by the 2019 coronavirus pandemic, new avenues of vaccine development have been opened, ones which are arguably easier to develop and more cost-effective. To ameliorate disadvantages in these platforms some groups have employed heterologous prime boost strategies to help carve a desired immunotype, thereby increasing protective responses. To this end, there is a solid foundation to explore novel vaccination strategies.

**Table 3:** Advantages and disadvantages of various vaccine platforms.

Vaccine Platform	Advantages	Disadvantages	Key Features
Irradiated Parasite	Strong protection, especially with increasing doses	Heterogenous vaccine Parasite life-cycle dependent for development Unethical as some parasite may mature to adult stage	Mimic natural infection
Adjuvanted Protein	Various adjuvants will give different immune responses Shown to be effective Can be used in populations with weakened immune systems	Can be expensive to produce	Immune response varies depending on adjuvant used
Nucleic Acid (DNA based)	Quick and simple design Thermostable Cost effective	Low immunogenicity in humans Enters host cell nucleus	Mostly cell mediated immune responses
Viral Vectored	Specific delivery of antigen to target cells High antigen expression Gene expression can be short or long term	Neutralizing immunity Off target virus shedding	Humoral responses CD8+ T cell responses Th1 dominant CD4+ T cell responses

Helminths have evolved with humans and in this time have developed ingenious methods of evading our immune response. To create an effective, protective response against helminths we must counteract these evasion mechanisms by developing vaccines which harness correlates of immunity. There has yet to be a human helminth vaccine approved by any regulatory agency but considering the constructs currently in clinical trials and the promising vaccines in preclinical development, this may change in the near future.

## Author Contributions

This review was written by DP and edited by MN. All authors contributed to the article and approved the submitted version.

## Funding

This work was supported by grants provided by the Public Health Agency of Canada, Canadian Institutes of Health Research (FBD-175968), and the Foundation of the McGill University Health Centre.

## Conflict of Interest

The authors declare that the research was conducted in the absence of any commercial or financial relationships that could be construed as a potential conflict of interest.

## Publisher’s Note

All claims expressed in this article are solely those of the authors and do not necessarily represent those of their affiliated organizations, or those of the publisher, the editors and the reviewers. Any product that may be evaluated in this article, or claim that may be made by its manufacturer, is not guaranteed or endorsed by the publisher.
